# Scientific Items

**Published:** 1881-08-20

**Authors:** 


### SCIENTIFIC ITEMS.

**Microscopic Examination of Blood.**—The characteristic distinctions of human blood and those of the blood of other animals are thus set forth in No. 2 of L'Orosi by Dr. Vincenzo Peset y Cervera, and if other physiologists will take the pains to verify his work, they may play a very important part in medical jurisprudence. When blood is mixed with a little bile, small crystals not over 0.003 of a metre in size are formed; but these crystals, the Doctor says, will show whence the blood had come. If from man, the crystals will be rectangular prisms; if from the horse, they will be cubes; if from the ox, they will be rhombohedrons; if from the sheep, they will be rhombodric tablets; if from the dog, they will be rectangular prisms, closely resembling the human forms; if from the rabbit, they will be tetrahedrons; if from the squirrel, they will be hexagonal tablets; if from the mouse, they will be octahedrons; and if from common poultry, they will be cubes more or less perfect. There would seem to be room for more accurate research in this direction.—*Drug. Circular.*

**Transatlantic Cables and their Life.**—The life of a submarine telegraph cable is from ten to twelve years. If a cable breaks in deep water after it is ten years of age, it cannot be lifted for repairs, as it will break of its own weight, and the cable companies are compelled to put aside a large reserve fund in order that they may be prepared to replace their cables every ten years. The action of the sea water eats the iron wire completely away, and it crumbles into dust. while the core of the cable may be perfect. The breakages of cables are very costly, and it is a very difficult matter to repair them, in comparison with land lines. A ship has to be chartered at an expense of $500 a day for two or three weeks in fixing the locality and in avoiding bad weather, as cables can only be repaired in the calmest seasons. One break alone in the Direct Company's cable cost $100,000 to repair, and the last chance left to the company was to make an agreement with the Anglo-American, so that they should be protected and have the use of that company's line when their own was stopped.—*Ibid.*

**Providence of Bees.**—The Melbourne correspondent of the Dundee Advertiser narrates the following interesting proof of the provident and far-seeing instinct of bees: Turning from men to insects, a singular circumstance is reported from a hot dry valley in New South Wales. Last year the drought there was of long duration, and the denizens of the apiaries suffered much from it. This year the bees have made provision against a similar emergency. They have filled a large number of the external cells in every hive with pure water instead of honey. It is thought that the instinct of the little creatures leads them to anticipate a hot summer. But that they should have gone further, and, by an act which, as far as I know, is without precedent in the habits and customs of their tribes, have created reservoirs to tide over the water famine, is a noteworthy fact indeed.

**Electrical Science.**—It is difficult to foresee into what functions of civilized life electricity is destined to enter; or rather, it would be hard to name any from which it will be excluded. Already we talk, write and travel by means of it; our streets and homes are illuminated by it, and it is an invaluable ally of the mechanician, the physician and the surgeon. We speak of it as a motor and an illuminating agent, although both of these applications of electricity are as yet in the experimental stage. Of their success and ultimate wide utility, however, there can no more be a resonable doubt than of the progress toward perfection of any other of the useful arts. Whether it may not afford the solution of still another formidable problem—the heating of interiors of houses, and in general the supply of heat for domestic and mechanical purposes—is a question not yet practically investigated; but there are evident reasons assignable why it may prove to be, if not a source of heat, at least a serviceable medium for its transmission. The recent triumphant success of the experiments made in the storeage of electricity by M. Faure, the French scientist, who was able to send a million foot-pounds of electrical energy from Paris to Glasgow, snugly packed in a box, prepare us for any extreme of daring or eccentricity on the part of inventors in this field.

With the new applications of electricity come manifold new dangers, which, however, will no doubt be easily removed when ascertained. The electric railway in Berlin developed an unforeseen propensity for shocking horses through the iron of their shoes when brought in contact with the rail. One horse fell down paralyzed, while others ran away in wild terror. In an eastern manufactory, not long since, an electric machine used for furnishing light for an adjacent store drew to its magnetic revolving armature a pair of scissors out of a man's hand, and its own wires being cut by the broken and flying scissors the electrict current escaped from the fractured ends, and the room, as an exchange describes the scene, was "filled to the ceiling with whirling lightning." Happily, no one was injured; but it was a revelation of the terrible power which this fleet servant of mankind possesses—power not only to serve, but also to rend and kill.—*Lef. Mech. News.*

**Bronzing Liquid.**—

| | |
|---|---|
| Aniline red | 10 drachms. |
| Aniline purple | 5 " |
| Alcohol of 95 degrees | 12½ ounces. |
| Benzoic acld | 5 drachms. |

Dissolve the aniline colors in the alcohol over a water bath, then add the benzoic acid, and boil the mixture for five or ten minutes, until the green color has turned to a light brown. The liquid, on being applied to metals, leather, wood, etc., exactly imitates bronze,

**To Remove Rusted Bolts.**—To remove bolts that have rusted in, without breaking them, the most effective remedy that we know of, is the liberal application of petroleum. It rarely fails to accomplish the work, Care must be taken that the petroleum shall reach the rusted parts, and some time must be allowed to give it a chance to penetrate beneath and soften the layer of rust, before the attempt to remove the bolt is made.—*Ibid.*

